# Artificial Intelligence-Assisted Endoscopic Assessment of Response to Neoadjuvant Chemoradiotherapy in Esophageal Cancer: From Residual Disease Detection to Active Surveillance and Surgical Decision-Making

**DOI:** 10.3390/diagnostics16213598

**Published:** 2026-10-31

**Authors:** Yinjie Tao, Xin Zhao, Yuda Zhao, Na Wang, Mengyu Wang, Fang Yao

**Affiliations:** Department of Endoscopy, National Cancer Center/National Clinical Research Center for Cancer/Cancer Hospital, Chinese Academy of Medical Sciences and Peking Union Medical College, Beijing 100021, China; taoyj15@tsinghua.org.cn (Y.T.);

**Keywords:** esophageal neoplasms, neoadjuvant chemoradiotherapy, artificial intelligence, active surveillance

## Abstract

Neoadjuvant chemoradiotherapy (NCRT) followed by esophagectomy remains standard for locally advanced resectable esophageal cancer. Active surveillance defers or avoids surgery in patients who meet clinical complete response (cCR) criteria. What that judgment rests on is fragile: cCR is assembled from whichever modality was used, at whatever time point, under whatever follow-up intensity. cCR is a clinical classification, whereas pathologic complete response (pCR) is established on a resection specimen. Endoscopy carries most of the weight, and observer-dependent interpretation remains a concern. We searched PubMed, Embase, and the Cochrane Library from inception to 15 July 2026 and synthesized the findings narratively. Treatment settings differ enough to distort any pooled reading, so we stratified the evidence into four tiers—endoscopic AI in NCRT-treated cohorts, separated by pretreatment versus post-treatment image acquisition, conventional endoscopic and pathological response assessment, indirect AI evidence from neoadjuvant chemotherapy or immunotherapy, and other clinical and methodological context—rather than pooling it. Prediction model and diagnostic studies were independently appraised by two physicians using design-specific tools. Active surveillance achieved non-inferior two-year overall survival compared with immediate surgery in the SANO trial. preSINO measured how partial that reassurance is: bite-on-bite biopsy with endoscopic ultrasound-guided fine-needle aspiration yielded a negative predictive value of 68.7% for residual disease. Among the seven endoscopy-based modeling reports summarized, one used pretreatment images in an NCRT cohort, and six were indirect studies from non-radiotherapy or predominantly non-radiotherapy cohorts. None separately validated AI on post-NCRT images for current residual disease detection. Most are retrospective, single-center, static image studies conducted in chemotherapy rather than chemoradiotherapy cohorts; the largest enrolled 1041 patients across 46 centers and reported 80% sensitivity and 90% specificity for primary site pCR, not patient-level ypT0N0. Patient-level sensitivity, negative predictive value, calibration, and decision curve analysis are seldom reported. Endoscopic AI belongs inside composite response assessment, as a second reader—not as the thing that decides whether surgery can be omitted or surveillance relaxed. The most informative study still unperformed is not another AUC comparison; it is a prospective trial adding AI-targeted sampling to systematic bite-on-bite biopsy, with the patient-level false-negative rate for residual disease as the primary endpoint. AI has the potential to standardize image interpretation; prospective reader studies are needed to establish reproducibility in practice. Whether it makes active surveillance safer is not yet known.

## 1. Introduction

Locally advanced resectable esophageal cancer is generally treated with neoadjuvant chemoradiotherapy (NCRT) followed by esophagectomy. Regression is often substantial, and in some patients no viable tumor is left in the specimen—a pathologic complete response (pCR). That observation has made active surveillance an option for patients who meet clinical complete response (cCR) criteria after standardized response evaluation, and has made it one of the main lines of investigation in organ preservation. Surveillance does not cancel surgery; it defers or avoids esophagectomy under intensive reassessment, with salvage surgery prompted by residual disease or regrowth. Its purpose is narrower than proving pCR without a specimen: to separate the low-risk patients who can keep being watched from the high-risk patients who need an operation now, and to do that without costing anyone a cure [1,2,3,4,5].

SANO (Surgery As Needed for Oesophageal Cancer), a multicenter, stepped wedge, cluster-randomized, non-inferiority phase 3 trial, provides the highest level of clinical evidence for this strategy. Screening 1115 patients across 12 Dutch hospitals yielded 309 who reached cCR after NCRT; 198 were managed by active surveillance and 111 by standard surgery. At a median follow-up of 38 months, two-year overall survival was 74% with surveillance and 71% with surgery, meeting the prespecified non-inferiority criterion. During surveillance, 83 patients proceeded to deferred esophagectomy for locoregional regrowth, at a median of 5.9 months after cCR. Positive margin rates, postoperative complication rates, and 90-day mortality were similar between groups, and global health-related quality of life at six and nine months after treatment favored surveillance. Given rigorous response evaluation and close follow-up, active surveillance earns its place as an option within shared decision-making. Two caveats travel with it: follow-up is still short, and locoregional regrowth is common. Making the strategy safe therefore depends on detecting residual disease and early regrowth better than we do now [6].

After NCRT, inflammation, edema, necrosis, ulceration, re-epithelialization, scarring, and deep fibrosis can coexist in the same segment of the esophageal wall. White light endoscopy (WLE) shows the mucosal surface, and a superficial biopsy misses submucosal or scattered residual tumor with uncomfortable ease. Endoscopic ultrasonography (EUS) reads wall thickness and layer structure, but post-treatment fibrosis blunts both. CT and PET-CT seldom exclude small locoregional residual disease. The cCR assembled from these tests is a clinical state, defined jointly by which modality was used, when it was used, and what follow-up later showed. It is not pCR by another name [7,8,9,10,11].

The distinction concerns the reference standard and the tissue that can be assessed. Endoscopy visualizes the mucosal surface; even negative biopsies sample only selected sites and may miss microscopic, submucosal, or discontinuous residual tumor. EUS is confounded by treatment-related wall fibrosis, although needle sampling can establish disease in suspicious nodes. CT and PET-CT assess extraluminal or systemic disease but cannot exclude microscopic local deposits. Surface image AI may improve recognition and sampling of visible abnormalities, but it cannot directly establish the absence of tumor in unsampled deep tissue or regional nodes. Whether AI assistance improves cCR classification requires prospective evaluation against an appropriate composite reference standard [7,8,9,10,11].

Earlier work provides a technological rationale rather than evidence of response assessment. Tsai et al. combined computationally reconstructed hyperspectral information from white-light and narrow-band endoscopic images with a deep learning detector for early esophageal cancer. This illustrates how optical information beyond conventional RGB representations may contribute to image interpretation; it did not evaluate post-NCRT response and is cited only as technical background [12].

Artificial intelligence (AI) offers a different route to the same problem. It extracts color, textural, morphological, and spatial heterogeneity features from endoscopic images or video—quantities that vary continuously and that no two observers score identically—and can put them to work on localization of suspicious areas and on risk models that update over time. Investigators have begun applying it to pretreatment and post-treatment endoscopic images to predict pathologic response. Within the studies summarized here, no model separately validates current residual disease detection using post-NCRT images or tests AI-assisted management during active surveillance. These applications remain research objectives.

**Figure 1:**
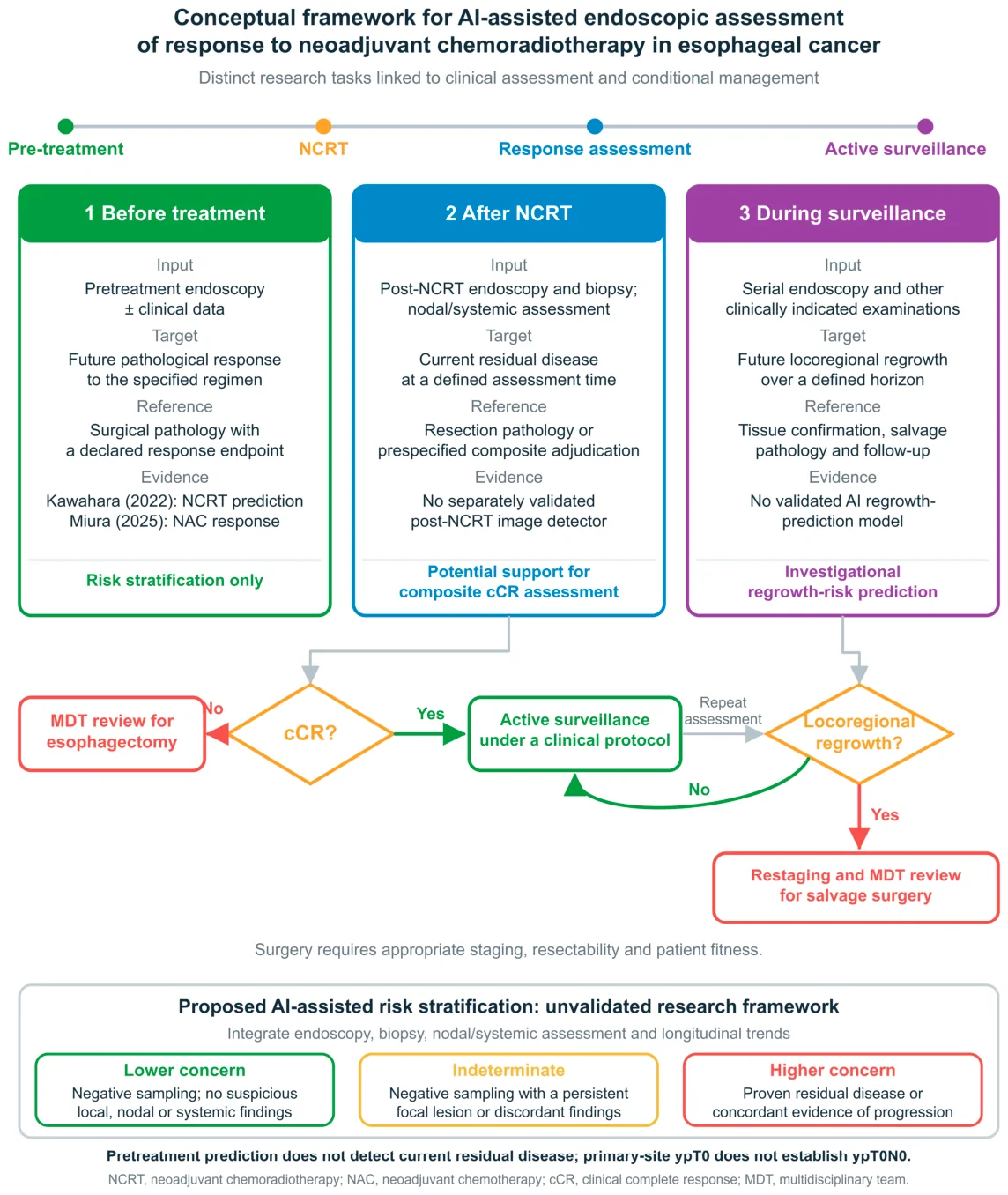
Conceptual framework for endoscopic AI across the treatment pathway. Pretreatment prediction, post-NCRT residual disease assessment and surveillance-regrowth prediction have distinct inputs, endpoints and reference standards. Kawahara [13] and Miura [16] illustrate pretreatment tasks in NCRT and NAC cohorts, respectively. AI contributions and the proposed risk strata are unvalidated; cCR assessment remains composite, and surgery requires restaging and multidisciplinary review. Primary site ypT0 does not establish patient-level ypT0N0.

This review takes endoscopy as its anchor and works through five things in order: the pathologic basis of residual disease after NCRT, the candidate endoscopic findings, what endoscopic AI has actually shown, longitudinal and multimodal modeling, and how any of it might be used in active surveillance and surgical decision-making [13,14,15,16,17]. Figure 1 sets out the three-stage framework that organizes what follows.

## 2. Literature Search, Evidence Stratification, and Key Concepts

The primary review question is structured as follows: in adults with resectable, locally advanced esophageal cancer treated with NCRT (population), can AI applied to standardized endoscopic images or video (index test), compared with endoscopist interpretation and standard clinical response evaluation (comparator), improve patient-level detection of current residual locoregional disease and the safety of active surveillance (outcomes)? The reference standard is resection pathology when available, or prespecified composite adjudication incorporating tissue confirmation, imaging, salvage pathology, and adequate follow-up. Pretreatment pathologic response prediction and future regrowth prediction are secondary questions with distinct time origins and reference standards; NAC/nCIT cohorts and non-endoscopic models provide indirect or methodological context.

We conducted a structured search of PubMed, Embase, and the Cochrane Library from inception to 15 July 2026, with backward screening of reference lists and narrative synthesis. Evidence was stratified into four categories: endoscopic AI in NCRT-treated cohorts, separated by image acquisition timing; conventional endoscopic and pathological response assessment; indirect endoscopic AI after neoadjuvant chemotherapy or immunotherapy; and other clinical and methodological context. Findings from other regimens or histologic types provide context rather than evidence for omitting surgery after NCRT [18,19].

Complete database-specific main and supplementary search strategies, with their record yields, are provided in Supplementary Methods S1. Two reviewers screened the records independently; disagreements were resolved by consensus or arbitration by a third reviewer.

The following eligibility criteria apply to the core response-assessment synthesis; additional clinical and methodological reports were retained as labeled context. Studies were eligible if (1) the study population comprised patients with pathologically confirmed esophageal cancer, including squamous cell carcinoma and adenocarcinoma; (2) patients received neoadjuvant therapy, including NCRT, neoadjuvant chemotherapy (NAC), neoadjuvant chemoimmunotherapy (nCIT), or total neoadjuvant therapy (TNT); (3) endoscopy (white-light endoscopy, image-enhanced endoscopy, EUS, or endoscopic biopsy) was used for response assessment, or AI, machine learning, or deep learning methods were applied to endoscopic images to predict treatment response; or (4) the study addressed an active surveillance strategy and salvage surgery. Also required were (5) a design that was a primary study (prospective or retrospective), a systematic review/meta-analysis, or a randomized controlled trial, and (6) publication in English with full text available. Studies were excluded if (1) the study did not concern esophageal cancer, although cross-cancer methodological studies were retained as technical references; (2) endoscopic assessment was not involved, although multimodal studies containing an endoscopic component were retained; (3) the study concerned only early endoscopic screening or diagnosis and not response assessment; (4) the publication was a conference abstract, letter, comment, editorial, or other report without complete data; (5) it was a case report; (6) it was not in English; or (7) it was a duplicate publication.

After deduplication, 104 of 348 database records underwent title/abstract screening; 42 were excluded. All 62 reports sought were retrieved and assessed in full, with 13 excluded (ineligible treatment setting, 3; outside the review topic, 9; non-article publication, 1), leaving 49 included reports. Reference-list screening added no included reports (Supplementary Figure S2).

During revision, the 49 selected reports were assigned once by their primary contribution: NCRT-cohort endoscopic AI (1), conventional response assessment and pathology (21), indirect endoscopy-based response models (6), and other clinical or methodological context (21) (Table S4). These categories describe relevance, not a formal certainty hierarchy or independent cohorts. Other technical or protocol citations are not included in these selection counts. The seven core endoscopy-based modelling reports comprise one NCRT pretreatment report and six indirect reports. None separately validates post-NCRT image-based residual detection or surveillance-regrowth prediction; EC-HAENet included three radiotherapy-treated patients without a separately validated NCRT subgroup.

Two physicians independently appraised full reports using PROBAST + AI (2025, model evaluation component) [20] for prediction model performance and QUADAS-2 for conventional diagnostic accuracy. One instrument was assigned per assessment; applicability was rated separately. The model group included seven core endoscopy-based reports and one additional routine clinical variable model. ROBINS-I was not performed. SANO had signaling question records for five standard RoB 2 domains but no final domain judgments, complete cluster-specific appraisal, or overall trial rating. Non-randomized management comparisons and background-only sources were not formally appraised. Supplementary Table S3 and Figure S1 report completed judgments; no composite score or quantitative quality weighting was used. Resource availability verification (Table S2) was conducted separately from formal risk-of-bias appraisal (Table S3).

The completed appraisal included 17 assessment rows from 16 reports (68 risk-of-bias and 51 applicability judgments); EUS and FNA estimates from the study by van der Bogt et al. [21] were assessed separately. Both physicians agreed on all judgments. All eight models had unclear analysis domain judgments. Among nine diagnostic assessments, four had high-risk patient selection, six had high-risk flow and timing, and all nine had unclear reference standard judgments. Counts describe report estimate assessments, not independent cohorts.

Four terms do most of the work in this review, and they are not interchangeable. Pathologic response covers the spectrum of regression graded on the resection specimen, often with a tumor regression grade (TRG). Patient-level pCR denotes ypT0N0; primary site pCR denotes ypT0 and leaves nodal status unresolved. Residual disease means viable tumor persisting at the primary site and/or regional nodes after treatment. A study-specific response threshold such as Grade 1b–3 is a broader endpoint than pCR. cCR is a time-specific clinical classification and means the absence of definite residual evidence at a defined time point across endoscopy, adequate biopsy, EUS, and systemic imaging. Sustained cCR extends that negative assessment across consecutive surveillance examinations once cCR has first been reached. Locoregional regrowth is the reappearance of confirmed or strongly suspected tumor at the primary site or in regional lymph nodes after an initial cCR. The three modeling tasks built on these endpoints—pretreatment prediction of treatment sensitivity or postoperative pCR, post-treatment detection of current residual disease, and prediction of future locoregional regrowth during surveillance—differ in reference standard, model architecture, and clinical consequence, and training them under a single label conflates all three. In the active surveillance setting, patient-level sensitivity, false-negative rate, negative predictive value, calibration, Brier score, and decision curve analysis matter at least as much as the area under the receiver operating characteristic curve (AUC), and reports need to give all of them.

The available studies differ too much—in regimen, timing of examination, mode of endoscopic acquisition, endpoint definition, and validation design—for quantitative pooling of model performance to mean anything. We therefore compare studies narratively, with the specific clinical task as the unit of comparison. In active surveillance cohorts, one negative endoscopy or one negative biopsy is not an adequate reference standard. A defensible adjudication combines endoscopy with systematic or targeted biopsy, EUS sampling of suspicious lymph nodes, cross-sectional or metabolic imaging, pathology from salvage treatment, and follow-up long enough to matter. Two reporting requirements follow from this: state how much follow-up a negative case needs before it can be classified as such, and state the arbitration rule applied when tests disagree. Both reduce reference standard bias [7,8,9,10,11,21,22,23,24,25,26].

## 3. Pathological Basis of Residual Disease After NCRT and Limitations of Conventional Assessment

Residual tumor after NCRT often fails to form a continuous, macroscopically visible mass. It appears instead as minute nests within the mucosa or submucosa, as scattered single cells, or in insular and skip distributions, and some deposits reach the muscularis propria or periesophageal tissue. Pathology series make the uncomfortable point directly: submucosal disease persists even after the mucosal surface has clearly improved or re-epithelialized. Surface phenotype and deep pathology diverge, and that divergence is where false-negative results in conventional endoscopic inspection and superficial biopsy come from [27,28,29,30,31].

Radiation esophagitis brings diffuse hyperemia, edema, erosion, and ulceration. The repair phase brings granulation tissue, scar contraction, and interstitial fibrosis, which read as elevation, pale patches, or stricture. Benign as they are, these changes overlap with residual tumor in color, surface irregularity, friability, and luminal deformity. One static finding shifts the probability of residual disease; it does not settle the question. The inflammatory and repair phases also differ between assessment time points, so an endoscopic AI model needs to be trained and validated inside a defined, reasonably consistent window.

A major endoscopic or pathologic response at the primary site says nothing about the regional nodes. Any model trained only on images of the primary tumor bed describes a mucosal surface and its immediate surroundings, and micrometastasis in regional nodes lies outside that field of view. A low-risk AI output therefore cannot be read as clearance of regional or systemic disease, which leaves EUS-guided sampling of suspicious nodes and systemic imaging in place [21,22].

Spatial heterogeneity also governs how clean the labels can be. Label every post-treatment image from a patient with a negative biopsy as “complete response”, and systematic label noise enters wherever deep or skip residual disease is present. Draw regions of interest only around visible mucosal abnormalities, and the model learns inflammation and fibrosis instead of tumor. The most direct route to a trustworthy model runs through composite reference standards—surgical pathology, positive biopsy, salvage surgery pathology, or adequate long-term follow-up—registered in space against the images, the biopsy coordinates, and the resection specimen.

## 4. Endoscopic Candidate Findings, Sampling Strategy, and Evidence for AI-Based Prediction

### 4.1. White-Light Endoscopic Findings and Their Quantitative Performance

White-light endoscopy is still where assessment of the primary tumor bed begins. The endoscopist’s overall judgment—is there definite suspicious residual disease here or not—carries real predictive value, though no subjective impression alone justifies omitting surgery. Persistent or new nodules, focal elevation, a granular surface, irregular depression, and focal mucosal abnormalities that stand out against the surrounding inflammatory background are best treated as priority sampling targets. They are not proof of residual tumor [32,33,34,35,36].

An ulcer means whatever its morphology and its behavior over time say it means. Marginal nodularity, focal elevation, progressive surface irregularity, or deterioration across successive examinations deserve more concern than the mere presence of an ulcer. Flat scar, complete re-epithelialization, and smooth mucosa usually signal good response, and they still leave room for minute submucosal residual disease. Stricture after treatment may be residual tumor or radiation fibrosis, and a single judgment about whether the scope can pass is poorly specific. A stricture that lengthens or tightens, particularly an eccentric one alongside a focal nodule or a deteriorating ulcer, warrants more intensive re-examination and sampling [33,36].

Studies [32,33,34,35,36] quantified post-treatment endoscopic information from several directions: structured endoscopic classification, overall judgment of suspicious residual disease, macroscopic tumor residue, biopsy, and multi-sign scoring. Their combined lesson concerns what to feed a model. Isolated binary variables—ulcer present, scar present, stricture present—carry little. Combined phenotypes carry more: residual mass, villous surface, focal traction, diffuse infiltrative or obstructive change, non-superficial lesion, abnormal intraepithelial papillary capillary loops (IPCL), uneven superficial erosion, and protrusion, each read alongside its evolution over time. Table 1 collects the quantitative evidence.

This body of evidence supports a hierarchy. Definite residual mass and obstructive or infiltrative change behave as high-specificity risk features. Residual mass, villous surface, focal traction, non-superficial lesion, abnormal IPCL, uneven erosion, and protrusion work better as localization features that decide where to sample first. Ulcer, scar, and stricture are background: they need morphological detail and longitudinal change attached before they mean much. None of this points toward reproducing one endoscopist’s binary judgment. The task worth attempting is to convert these multiscale findings into reproducible quantitative representations, then read out two numbers—patient-level residual risk, and region-level sampling priority.

### 4.2. Image-Enhanced Endoscopy and Magnifying Assessment of the Post-Treatment Mucosa

Narrow-band imaging (NBI), blue laser imaging (BLI), linked color imaging (LCI), and magnifying endoscopy all raise contrast in mucosal color and microvasculature, so in principle they ought to expose focal abnormalities against diffuse treatment-related inflammation. Candidate features include focally increased vascular density, uneven caliber, disordered course, and color asymmetry. One classification system is worth singling out because it is frequently borrowed without justification: the intraepithelial papillary capillary loop classification was developed for untreated early esophageal squamous cell carcinoma and cannot be carried over to the post-NCRT mucosa without validation, since radiotherapy causes vascular obliteration, remodeling, and chronic inflammation, all of which change what the loops look like. Direct evidence is insufficient. Treat these appearances as candidate findings awaiting validation, not as criteria on which to base a surgical decision.

### 4.3. Biopsy Strategy, the Role of EUS, and the Residual Risk Quantified by preSINO

Conventional superficial biopsy fails in two ways: wrong place, and not deep enough. preSANO and the work that followed it argue for thorough systematic sampling of the primary tumor bed and for bite-on-bite biopsy to reach deeper tissue, both of which lower the chance of missing submucosal residual disease. The strategy that follows pairs targeted biopsy of regions flagged by the eye or by AI with systematic biopsy of the tumor bed. The two are complements. Targeted sampling does not license dropping systematic sampling [23,24,25].

Fibrosis produces wall thickening and loss of layer structure after NCRT, which is why EUS restaging of T is inaccurate. EUS earns its place elsewhere: identifying suspicious regional nodes, and sampling them by fine-needle aspiration or fine-needle biopsy through a linear echoendoscope when that is technically feasible. That covers regional risk no amount of primary site endoscopy, or biopsy can reach. Routine puncture of every visible node is not the implication. Which nodes to sample, and when to escalate, belong to a standard clinical response evaluation protocol and to multidisciplinary discussion [21,26].

preSINO, a prospective multicenter study in East Asian patients with esophageal squamous cell carcinoma, performed CRE-1 with bite-on-bite biopsy 4–6 weeks after NCRT and CRE-2 with PET-CT, repeat biopsy, and EUS-FNA of suspicious nodes at 10–12 weeks in initially negative cases. Among 242 surgically verified patients, 169 had any residual locoregional disease (TRG2–4 or ypN+), a prevalence of 69.8%, and 73 had TRG1–ypN0. For this endpoint, sensitivity was 81.7% (138/169; 95% CI 75.1–86.8), specificity 93.2% (68/73; 84.9–97.0), NPV 68.7% (68/99; 59.0–77.0), and PPV 96.5% (138/143; 92.1–98.5). Thus, 31 of 99 negative evaluations still had some residual disease at surgery; this 31.3% post-test proportion is not an AI estimate or an individualized prediction. Separately, the primary high-risk endpoint (TRG3–4 or TRG1–2 with ypN+) occurred in 133/242 patients; 18/133 were missed, giving an FNR of 13.5% (95% CI 8.7–20.4). The two endpoint-specific denominators must not be mixed. EUS-FNA identified nodal disease in six of nine patients with TRG1–2/ypN+ missed by primary site biopsy. PET-CT detected interval metastases in 13/268 screened patients (4.9%) [22].

NPV depends on residual disease prevalence, sensitivity, and specificity: NPV = Sp(1 − p)/[Sp(1 − p) + (1 − Se)p], where p is prevalence. The 68.7% NPV therefore applies to the surgically verified preSINO cohort, not automatically to a later low-risk surveillance population. For an individual patient, a negative local evaluation reduces risk but does not establish pCR or remove the need for nodal/systemic assessment and repeat surveillance. The false-negative rate among diseased patients (1 − sensitivity) differs from residual risk among test-negative patients (1 − NPV).

An exploratory analysis of tumor-informed circulating tumor DNA (ctDNA—testing customized to each patient’s individual tumor mutation profile) found that adding ctDNA to local evaluation reduced the false-negative rate from 14.9% to 5.4%; the 13.5% figure above comes from the full 242-patient cohort and the 14.9% from the 132-patient ctDNA subgroup, so the two denominators differ. Systemic recurrence rates were 28.0% in ctDNA-positive patients and 5.3% in ctDNA-negative patients. Read this as exploratory. ctDNA is better placed to supplement assessment of systemic minimal residual risk than to displace local endoscopic and nodal evaluation [22].

### 4.4. Evidence from Endoscopic AI Models

Pretreatment endoscopic AI stratifies treatment sensitivity; it does not confirm current residual disease after NCRT. Miura et al. studied 170 patients with locally advanced esophageal squamous cell carcinoma, comparing clinical information, pretreatment endoscopic images, and a fused model for predicting pathologic response to neoadjuvant chemotherapy. AUC was 0.55 for the endoscopic model, 0.64 for the clinical model, and 0.77 for the fused model. On their own, pretreatment endoscopic images carry limited predictive information, and clinical priors lift performance. The treatment setting was neoadjuvant chemotherapy, so for NCRT this counts as indirect evidence [16].

Kawahara et al. retrospectively enrolled 98 patients with locally advanced esophageal squamous cell carcinoma who underwent surgery after NCRT, training a convolutional neural network on 72 and reserving 26 for testing. After wavelet filtering, the model predicted pCR with a mean AUC, accuracy, sensitivity, and specificity of 0.83, 80.6%, 79.8%, and 80.6%. This is evidence for pretreatment prediction in an NCRT-treated cohort, not for image-based residual disease detection after NCRT. Pretreatment mucosal appearances already contain features usable for predicting pathologic response. The sample is small and retrospective, with no independent external validation; the full report also contains inconsistent train/test counts, so the abstract’s 72/26 split and the reported cross-validation performance are identified as such [13].

Post-treatment images come closer to the question at the center of this review. Matsuda and colleagues have taken endoscopic AI after neoadjuvant chemotherapy from proof of concept to large multicenter samples, and the trajectory is worth reading in order. The single-center study included 193 patients, and AI identified endoscopic responders with a median sensitivity, specificity, positive predictive value, and negative predictive value of 60%, 100%, 100%, and 71%. The four-center study included 123 patients, and the model identified pCR with median sensitivity, specificity, positive predictive value, negative predictive value, and accuracy of 60%, 81%, 77%, 67%, and 70%. The subsequent nationwide study across 46 Japanese esophageal centers included 1041 patients, and the same figures for pCR detection rose to 80%, 90%, 89%, 82%, and 85%, exceeding the average performance of specialist endoscopists [14,15,17].

Yuan et al. built EC-HAENet on 300 patients with esophageal cancer and 7359 post-treatment endoscopic images. Patient-level accuracy was 0.93 in the development cohort and 0.93 in the temporally independent test cohort, higher thanthat of concurrent endoscopic biopsy. The study is named for total neoadjuvant therapy, yet most patients received chemotherapy or chemoimmunotherapy and only a small minority received preoperative radiotherapy, so what it largely offers is indirect evidence after non-radiotherapy treatment. The test data also came from a different time period at the same center, which cannot substitute for cross-center external validation [37]. Table 2 separates clinical tasks, endpoints, and reference standards. Supplementary Tables S1A and S1B provide technical characteristics, and Table S2 reports reproducibility resources and verification limits.

**Figure 2:**
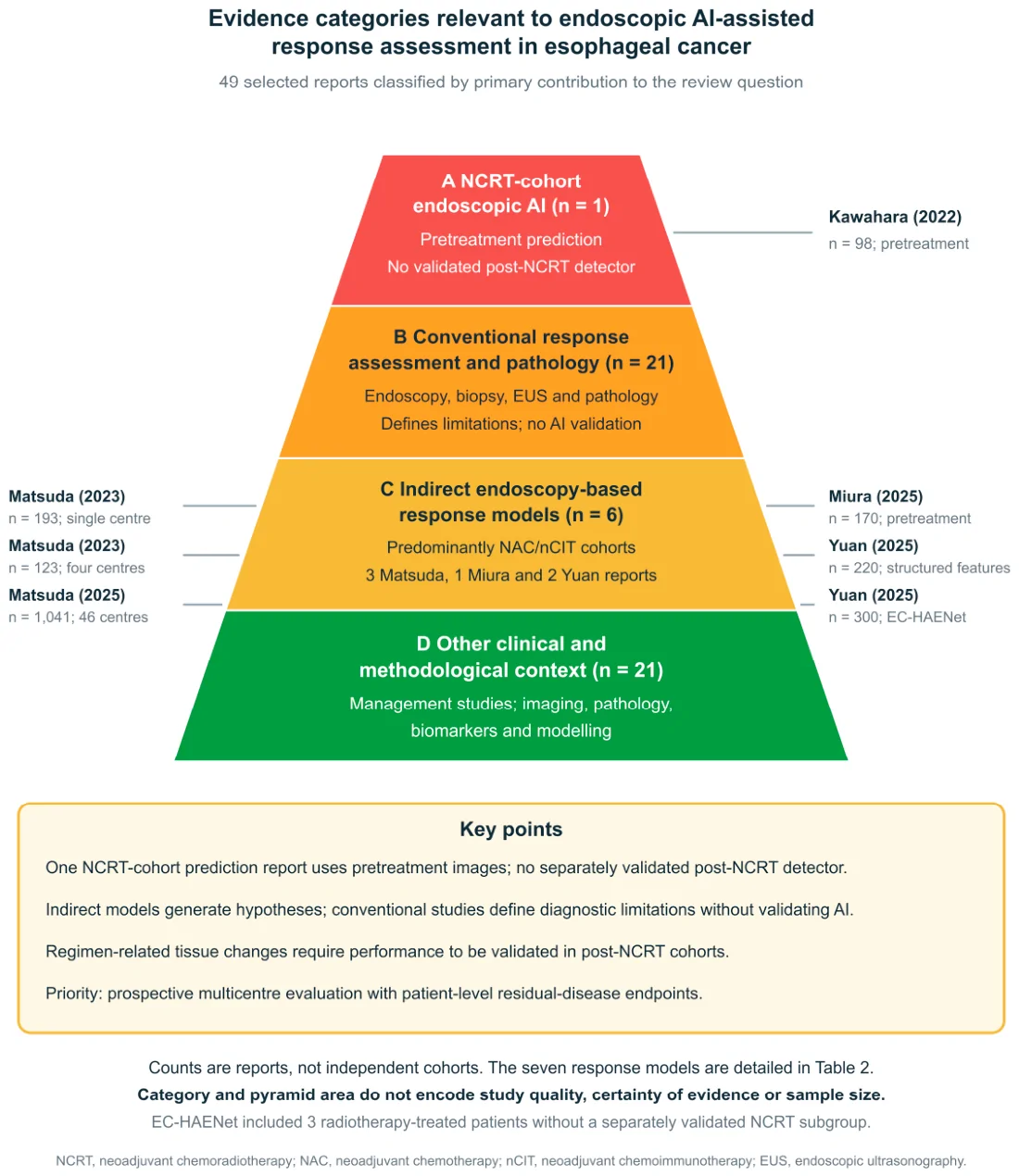
Evidence categories among the 49 selected reports: (**A**) one NCRT-cohort endoscopic AI report report using pretreatment images (Kawahara [13]); (**B**) 21 conventional response-assessment or pathology reports; (**C**) six indirect endoscopy-based response models (Matsuda [14,15,17], Miura [16] and Yuan [36,37]); (**D**) 21 other clinical or methodological reports. Each report is assigned once by primary contribution (Table S4). The pyramid is schematic, not a certainty or sample-size hierarchy; reports may share cohorts. None separately validates post-NCRT image-based residual detection.

The apparent performance of NAC/nCIT models cannot be transferred quantitatively to NCRT. Radiation-related vascular remodeling, inflammation, ulceration, and fibrosis can change both the input distribution and the relation between a visible feature and viable tumor. Representative image selection and surgically verified cohorts further differ from consecutive surveillance examinations. Calibration, operating thresholds, failure rates, and patient-level sensitivity must therefore be re-estimated in post-NCRT cohorts, rather than by carrying over a reported AUC or NPV. On this basis, the six indirect reports are hypothesis-generating; conventional post-NCRT pathology and diagnostic studies define the clinical detection problem, but do not validate an AI solution. Figure 2 summarizes these evidence categories.

Appraising these studies means keeping four levels of analysis apart: patient-level classification, image-level classification, weakly supervised localization, and lesion-level localization verified against pathology in space. A class activation map shows where the model attended and nothing more; it is not pixel-level tumor segmentation, and it is not biopsy navigation. Static image studies also lean on endoscopists to select representative high-quality images, and once several images from one patient enter both training and test sets, patient-level data leakage follows. Split training, validation, and test sets by patient. Report the image selection procedure, the number of images per case, and patient-level diagnostic performance. At this stage, AI reads better as a second reader, a risk alert, and a possible guide to where to sample than as an independent replacement for the endoscopist, the biopsy, or the multidisciplinary decision.

### 4.5. Levels of Analysis, Static Images Versus Full-Procedure Video, and External Generalizability

Static images selected by endoscopists are what most response prediction studies use. They store and annotate easily, and they discard the overall extent of the lesion, the transitions between near and far views, luminal traversability, and within-lesion heterogeneity, all while importing “representative image” selection bias. Full-procedure video keeps viewing angle, distance, and lighting, along with the continuous transition from lesion to surrounding mucosa, and so resembles a real examination more closely. Three methods make video tractable without frame-by-frame labeling: weakly supervised learning, which trains on image-level labels rather than pixel-by-pixel annotation; key frame selection, which chooses the most informative frames automatically; and temporal aggregation, which combines information across frames into one prediction. Video brings its own artifacts—runs of adjacent duplicate frames, motion blur, specular reflection, mucus obscuration, and camera instability. Data should be split by patient or by complete examination, and adjacent frames from one examination should be excluded from both training and test sets [38,39,40,41].

Generalizability beyond the development set rests on equipment and operating procedure as much as on the algorithm: endoscope manufacturer and processor, white-light or image-enhanced mode, resolution and compression format, viewing distance and angle, luminal insufflation, mucosal cleanliness, bleeding, and motion artifact. Severe post-treatment stricture can also prevent complete inspection of the tumor bed, and every technically difficult examination dropped from a dataset pulls the study population away from the real clinical one—spectrum bias by exclusion. Reporting needs device metadata, image quality exclusion criteria, and the proportion of examinations that were complete. Validation needs to be performed across manufacturers, across centers, and in consecutively enrolled patients rather than only on curated high-quality images [38,39,40,41].

## 5. Longitudinal Endoscopic AI and Endoscopy-Centered Multimodal Integration

A single post-treatment endoscopy depends on when it was done, how much inflammation was present, and which frames were kept. Longitudinal models escape some of these limitations by comparing lesion area, ulcer healing, nodule change, stricture velocity, and microvascular abnormality across baseline, the first post-NCRT assessment, the second assessment, and successive active surveillance visits, thereby assembling a response trajectory that belongs to that patient. For active surveillance, the direction of travel usually matters more than any single sign: is the endoscopic appearance still improving, holding steady, or deteriorating again? Complete endoscopic video also preserves what still frames are liable to lose, including the overall extent of the lesion, endoscopic passability, contact bleeding, and examination quality, but it requires solutions to inconsistent equipment, operators, viewing angles, insufflation, and localization of the primary tumor bed.

Longitudinal work on cross-sectional imaging offers indirect methodological support for modeling endoscopic time series. In a two-center study of 257 patients with esophageal squamous cell carcinoma receiving definitive concurrent chemoradiotherapy, a longitudinal deep learning model built on pre- and post-treatment contrast-enhanced CT achieved a concordance index (C-index) of 0.734 for overall survival in the external test set. A three-center study that combined pretreatment CT radiomics with clinical features to predict two-year locoregional recurrence reported a C-index of 0.744 in the validation set. Both point the same way: dynamic change may carry more information than a single time point. Neither population received NCRT, and neither endpoint was current residual disease after NCRT, so neither can serve as direct evidence for active surveillance [42,43].

The purpose of multimodal integration is not to add up positive findings. Each examination has blind spots, and the point is to let one modality cover another’s. Endoscopy shows the mucosal surface and the local phenotype of the primary tumor bed. Contrast-enhanced CT adds the esophageal wall, extraluminal invasion, and lymph node anatomy. PET-CT is used chiefly to detect interval distant metastasis and metabolically active suspicious nodes. MRI, especially diffusion-weighted imaging and the apparent diffusion coefficient (ADC), provides information on tissue cell density and diffusion. Liu et al. enrolled 151 patients at three institutions and fused conventional and deep radiomics features from pretreatment CT, T2-weighted imaging, and diffusion-weighted imaging; the test cohort reached an AUC of 0.868, with sensitivity of 88.0% and specificity of 78.4%, outperforming single-modality and clinical models [44]. Pre- and post-treatment CT deep radiomics combined with hematological indices, MRI radiomics combined with dynamic hematological indices, and PET radiomics combined with HER2 and CD44 expression likewise point to complementarity across information sources [45,46,47,48].

Pathology and molecular information can supply biological priors for the phenotypes endoscopy and radiology see. Immune features in pretreatment biopsies correlate with pathologic response to NCRT, and ctDNA may capture minimal residual disease or systemic risk that endoscopy cannot see. Digital pathology studies indicate that the spatial arrangement of tumor cells, cancer-associated stroma, and lymphocytes carries information about response to chemoradiotherapy and about survival. In the neoadjuvant chemoimmunotherapy setting, a model fusing CT radiomics with whole-slide pathology has already undergone multicenter external validation, and serum metabolomic and proteomic models have shown some predictive ability in patients treated with nonsurgical concurrent chemoradiotherapy. Their treatment contexts, endpoints, and data sources differ from post-NCRT active surveillance, so they should be read as indirect support for fusion architecture and biological interpretation [48,49,50,51,52,53].

Studies in other tumor types offer methodological lessons of the same kind. An eight-center study in locally advanced rectal cancer fused pretreatment MRI with endoscopic images through a ResNet–Vision Transformer and obtained an external test AUC higher than that of MRI or endoscopy alone, showing that endoscopy and cross-sectional imaging can be technically complementary. Disease site, endoscopic phenotype, and response pattern all differ, so the result cannot be carried over to esophageal cancer. What does this transfer mean? The implication of this transfer is that multimodal models in esophageal cancer should place endoscopy at the center and should demonstrate the incremental value of each additional modality over endoscopy alone rather than report only the AUC of the fused model [54].

Multimodal esophageal cancer research to date has combined CT, MRI, PET-CT, clinical variables, hematological indices, or digital pathology, with endoscopic images conspicuously underused. Limited data access is part of the explanation, but the deeper reason is structural. Endoscopy yields two-dimensional images or video whose field of view shifts continuously with lens position, angle, and insufflation, whereas CT and MRI sit in a comparatively standardized three-dimensional coordinate system, and the two generally have no reliable pixel-to-voxel correspondence. Endoscopic, radiological, pathological, and treatment data also tend to sit in separate information systems with inconsistent patient identifiers, storage formats, and examination time points, which further obstructs assembly of complete datasets [39,41,42,43,44,46,47,48,51,52,53,54].

Chasing precise spatial registration that the data cannot support is the wrong ambition for endoscopy–imaging fusion in esophageal cancer. Patient-level or lesion-level alignment gets further. Coarse localization can be established from the distance to the incisors, the longitudinal extent of the lesion, the circumferential proportion, the esophagogastric junction, and other anatomical landmarks, after which each modality is encoded independently and combined at the feature level or the decision level. Temporal alignment matters just as much. Pretreatment models may draw only on information available before treatment begins; post-treatment models need to state the interval from the end of NCRT to the endoscopy, CT, MRI, or PET-CT; longitudinal models must accommodate irregular intervals, missing modalities, and intervening interventions. Evaluation should report endoscopy alone, each other modality alone, and the fused model, and should use ablation analysis to establish the incremental contribution of each input, so that model complexity does not stand in for clinical utility.

## 6. From Response Prediction to Active Surveillance and Surgical Decision-Making

A pCR model built in a surgical cohort answers a conditional question: if this patient is resected, does the specimen meet the model’s specified response endpoint—primary site ypT0, ypT0N0, or a broader regression grade? Active surveillance asks something harder—to exclude current residual disease as safely as possible while no contemporaneous surgical pathology exists, and to keep detecting locoregional regrowth over time. Even a high predicted pCR probability must therefore pass through standardized cCR assessment, adequate biopsy, regional lymph node evaluation, systemic imaging, and multidisciplinary discussion before it can inform management, and it cannot be converted directly into a recommendation to omit surgery.

The SANO trial defines the clinical outcomes that AI will ultimately have to serve. In the active surveillance arm, 83 patients underwent deferred esophagectomy for locoregional regrowth, and the median time from achieving cCR to surgery was 5.9 months; R1 resection rates were 2% in both the active surveillance and standard surgery groups; R2 rates were 1% and 0%; postoperative complication rates were 82% and 84%; 90-day mortality was 4% and 5%. Under structured surveillance, deferring surgery until isolated locoregional regrowth appeared did not appreciably reduce the chance of radical resection or increase perioperative risk. AUC is the wrong yardstick for what comes next. What matters is whether AI can identify regrowth earlier, before distant dissemination, while preserving an R0 resection rate and a safety profile comparable to standard surgery [6].

Extended follow-up of the SANO cohort shows how little conventional clinical variables can do with this triage. The analysis expanded the cohort from the 309 patients with cCR enrolled in the main study to all 750 patients who completed NCRT and entered response evaluation, of whom only 274 (37%) had achieved cCR at 12 weeks, with the remainder reaching cCR at later assessments and 309 eventually randomized. Among the 198 patients who entered active surveillance, after a median follow-up of 54 months, only 49 (25%) maintained sustained cCR, 99 (50%) developed locoregional regrowth, 37 (19%) developed distant metastasis without locoregional regrowth, and 13 (7%) died without recurrence. Higher baseline cN stage was associated with a lower probability of initial cCR (cN2–3 versus cN0: OR 0.57) and a higher risk of non-sustained cCR (HR approximately 2.0), yet the optimism-corrected c-statistic of the corresponding models was only 0.56. Age, stage, histology, tumor location, and tumor length do not, in combination, identify who is genuinely suited to long-term active surveillance, and this gap is the direct clinical argument for quantitative endoscopic phenotypes and longitudinal AI [55].

A retrospective watchful waiting cohort of 50 patients reached a similar conclusion from the other direction: 30 patients (60%) recurred after a median of 202 days, 15 with locoregional and 10 with distant recurrence. Pretreatment endoscopy showing tumor involving more than 50% of the esophageal circumference was associated with recurrence (univariable OR 5.71; OR 25.19 after adjustment for age, sex, BMI, comorbidities, and stage), as was adenocarcinoma histology (univariable OR 3.92; adjusted OR 5.49), and dysphagia and fatigue at reassessment also pointed to higher risk. After multivariable adjustment, only adenocarcinoma remained statistically significant, with very wide confidence intervals, so these findings should be treated as hypothesis-generating. Pretreatment circumferential extent, symptoms at reassessment, and longitudinal endoscopic change may complement image phenotypes, but they need confirmation in larger multicenter active surveillance cohorts before they are built into AI models [56].

One probability of complete response cannot carry a treatment decision. An adequate model should output four separate risks: current residual disease at the primary site, current regional lymph node residual disease, locoregional regrowth within a defined period, and distant metastasis. Patients can be divided conceptually into three strata.

1. Low risk: no definite suspicious lesion at endoscopy, negative adequate biopsy of the primary tumor bed, negative sampling of suspicious regional nodes, no progression on systemic imaging, and a longitudinal risk estimate that is falling steadily.
2. Intermediate risk: negative biopsy but persistent focal abnormality, progressively worsening stricture, a rising AI risk score, or disagreement between modalities.
3. High risk: pathologically confirmed residual disease, positive regional node sampling, or concordant multimodal evidence of locoregional progression across consecutive assessments.

This stratification is a research framework. No fixed threshold within it has been validated prospectively.

**Figure 3:**
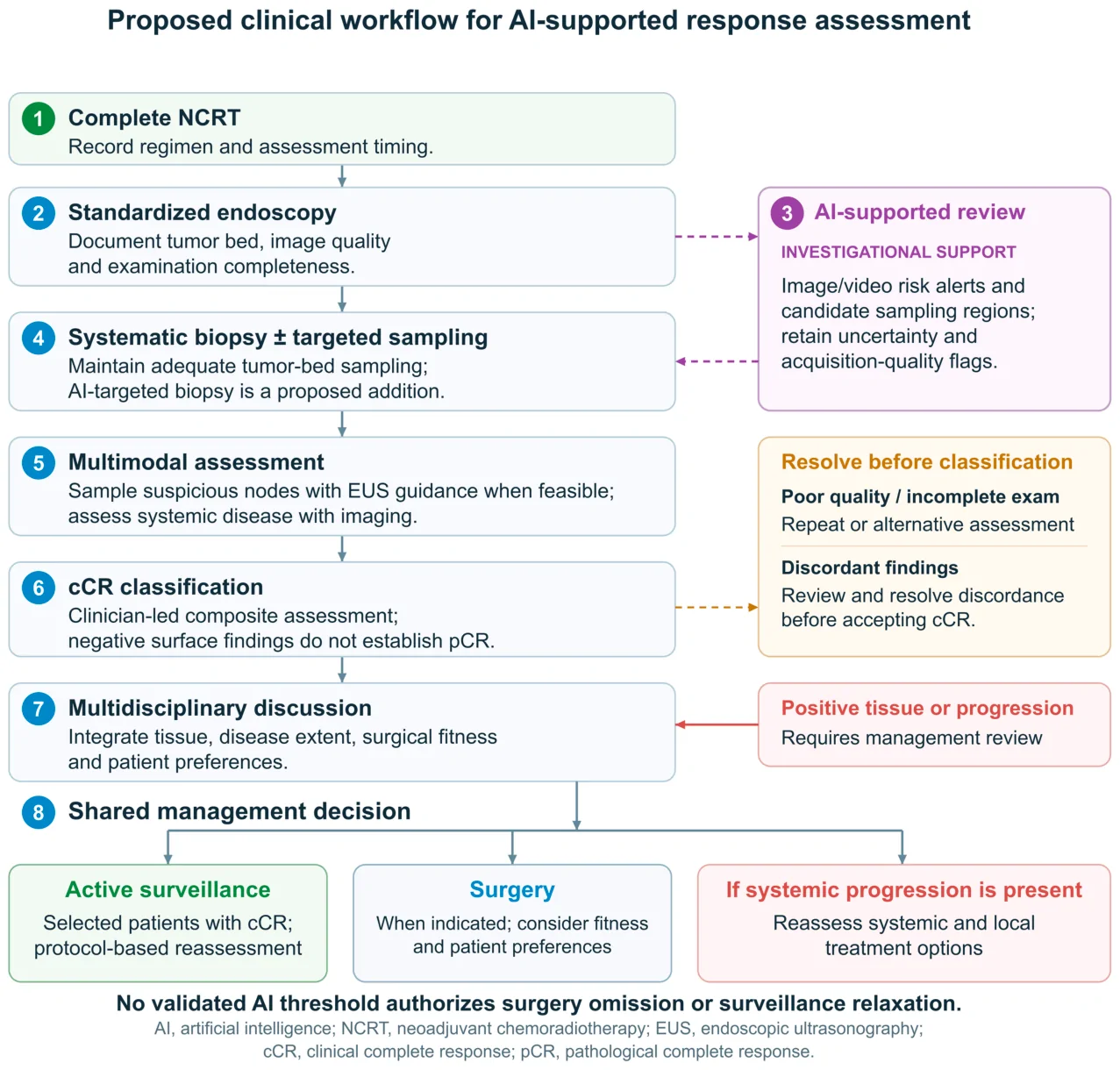
Proposed clinical workflow for AI-supported response assessment. The eight steps link NCRT to standardized endoscopy, AI-supported review, systematic with possible AI-targeted biopsy, multimodal assessment, cCR classification, multidisciplinary discussion, and surveillance or surgery. AI is an investigational decision-support input; sampling, cCR adjudication and management remain under clinical responsibility. Discordant findings, incomplete examination, and systemic progression require review rather than automatic surgery or surveillance. The AI contribution has not been validated as a basis for omitting surgery.

To connect risk outputs with action, the intended workflow begins with NCRT and standardized endoscopy, adds AI-supported image/video review and systematic biopsy with possible AI-targeted sampling, and then integrates nodal/systemic evaluation, cCR classification, multidisciplinary review, and shared decisions about surveillance or surgery (Figure 3). A low AI score does not cancel a positive biopsy, suspicious node evaluation, or a technically incomplete examination. Discordance or poor acquisition quality calls for clinical review, repeat/alternative sampling, or further assessment rather than an automatically favorable classification. Confirmed local or nodal disease requires staging and multidisciplinary management; distant progression changes the treatment objective.

No included study establishes a sensitivity or NPV threshold that safely authorizes deferral of potentially curative surgery after NCRT. Threshold selection should prioritize patient-level residual disease sensitivity, with prespecified confidence bounds and a clear definition of which residual lesions count as failures. The preSINO design’s maximum FNR of 19.5% for its high-risk diagnostic endpoint is not an acceptable AI threshold for surgery omission. An illustrative development target such as FNR ≤5% would be a proposed research criterion, not a validated clinical recommendation, and would require justification against standard assessment, delayed detection, salvageability, and patient preferences. Even zero misses among 60 diseased patients would only place the approximate one-sided 95% upper FNR bound near 5% (rule of three); this sample-size illustration does not establish safety. A prospective impact study must also assess R0 salvage resection, distant progression, survival, and examination burden.

Reducing the number of esophagectomies is only part of what active surveillance has to achieve; patients must still have a chance of R0 salvage resection when locoregional regrowth is found. Subsequent SANO analyses indicate that roughly half of locoregional regrowth occurred within the first three months of follow-up, that is, about six months after the end of NCRT, which makes the early period after initial cCR the critical window for intensified surveillance and dynamic risk updating [55]. Clinical utility should be measured as the number of non-beneficial surgeries avoided, the number of residual lesions missed, the lead time gained in detecting regrowth, the burden of repeated examinations, the complications of deferred surgery, and the R0 salvage resection rate. When distant metastasis or systemic progression is already present, a high local AI risk should not automatically trigger esophagectomy; systemic and local treatment should be re-evaluated. Age, comorbidities, surgical risk, quality of life, and the patient’s preference for organ preservation all belong in shared decision-making [1,2,3,4,5,6,55,56].

## 7. Methodological Limitations and Future Research Directions

This review was not prospectively registered, and the evidence categories were assigned during revision rather than prespecified; this may introduce subjectivity into evidence organization.

The central evidence gap is temporal as well as therapeutic: none of the seven endoscopy-based modeling reports separately validate detection of current residual disease using images obtained after NCRT. Kawahara’s NCRT cohort used pretreatment images; the three radiotherapy-treated participants in EC-HAENet do not constitute a separately validated post-NCRT cohort. Consequently, this review maps potential applications and validation requirements; it cannot establish diagnostic performance, reproducibility, or clinical benefit of endoscopic AI in post-NCRT surveillance.

The available studies are shaped jointly by heterogeneity in treatment regimen, histologic type, examination timing, and endoscopic equipment. The mucosal background differs after neoadjuvant chemotherapy, NCRT, immunochemotherapy, and total neoadjuvant therapy, and lesion morphology and regression patterns are not identical in esophageal squamous cell carcinoma and adenocarcinoma. Pooling these groups without stratifying by regimen and histology weakens both the clinical interpretability of a model and the safety of extrapolating it. Active surveillance cohorts add a further problem, because without contemporaneous surgical pathology the absence of detectable tumor over a short interval does not prove the absence of residual disease.

Endoscopic AI response research is also geographically and demographically concentrated, with most work coming from East Asia and dominated by ESCC. Esophageal adenocarcinoma differs in lesion location, background mucosa, endoscopic phenotype, molecular biology, and treatment pathway, so a model built in a squamous cell carcinoma cohort cannot be applied to adenocarcinoma without independent validation. Equipment manufacturers, image selection practices, NCRT regimens, response assessment, and surveillance workflows also vary across countries and centers. Future studies should report histologic subtype and geographic composition explicitly and should perform stratified validation across technical platforms, treatment regimens, and populations [14,15,16,17,18,19,38,39,40,41]. This also limits combining the two evidence streams: AI response-model cohorts are predominantly ESCC, whereas SANO predominantly enrolled adenocarcinoma. SANO evaluates a complete surveillance strategy, not an AI index test. Its clinical non-inferiority result cannot validate an ESCC-trained AI model, and the AI performance estimates cannot be imported into the SANO population. Histology-specific calibration and external validation are required within the intended NCRT and surveillance pathway, including an adequately represented adenocarcinoma cohort [6,13,14,15,16,17,22].

Potential sources of optimism include selected static images, patient overlap between training and test sets, and inadequate handling of class imbalance. Internal validation alone and AUC-only reporting limit evidence for transportability and clinical utility. Future work should partition datasets by patient and by complete examination so that images from the same patient never span training and test sets, and should report sensitivity, false-negative rate, negative predictive value, calibration, decision curve analysis, and clinical net benefit alongside AUC. Interpretability claims deserve the same skepticism as performance claims. A class activation map shows that the model attended to a region; it does not show that the region contains residual tumor, and claims about interpretability need verification through spatial mapping to biopsy or resection specimens.

Domain judgments informed narrative interpretation without numerical weighting. All eight models had unclear analysis domain judgments; reported performance was therefore treated as preliminary. Outcome-enriched Matsuda test samples limit transportability of predictive values, while the 20-image tests are imprecise. The structured Yuan model selected its cut-off using combined cohorts; EC-HAENet used a patient-level temporal split without center-held-out or dedicated post-NCRT validation. High-risk patient selection and flow and timing limit generalization of diagnostic estimates; unclear reference standard judgments retain uncertainty despite surgical pathology. Unclear ratings were not treated as low risk, and bias was distinguished from post-NCRT applicability. Incomplete appraisal of SANO and non-randomized management comparisons further limits confidence in the synthesis. Resource transparency is an additional limitation (Table S2), evaluated separately from risk of bias. A request-based data statement is not an openly downloadable dataset, and a published architecture is not an executable pipeline. Public code, trained weights, preprocessing, label definitions, and patient-level split identifiers should be distinguished.

A standardized endoscopic database should fix the key examination time points, store complete white-light and image-enhanced endoscopic video, and record the location of the primary tumor bed, lesion extent, endoscopic passability, the number and coordinates of biopsies, EUS-suspicious lymph nodes, and pathology results. It should also build a standardized dictionary and image atlas for nodules, ulcer, scar, re-epithelialization, stricture, bleeding, color, and microvascular abnormality. Study design is best organized around two cohorts. The surgical pathology cohort trains the current residual model on ypT, ypN, tumor regression grade, and the spatial distribution of residual disease; the active surveillance cohort validates the dynamic risk model against sustained cCR, time to locoregional regrowth, salvage surgery, R0 resection, and distant progression.

At the level of image acquisition, a minimum research specification is feasible. Before the examination, the location of the primary tumor bed should be defined; during it, a panoramic view of the tumor bed, a close-up view, and the proximal and distal margins should be saved in sequence, with image-enhanced endoscopy added whenever technically possible; the lesion should be observed repeatedly from different angles and distances, and the distance to the incisors, lesion length, circumferential proportion, endoscopic passability, and biopsy coordinates should be recorded in structured form. Endoscope and processor model, imaging mode, resolution, compression method, and enhancement parameters should be recorded as well. Quality control should flag rather than simply discard examinations with severe blur, excessive reflection, obscuration by mucus or blood, unclear anatomical location, or incomplete visualization of the lesion, since flagging both supports algorithm training and allows the failure risk of the model in technically difficult cases to be measured [38,39,40,41].

A prospective trial of AI-assisted biopsy could compare conventional systematic sampling with AI-targeted plus systematic bite-on-bite biopsy, with primary endpoints of patient-level residual detection rate, false-negative rate, number of biopsies per patient, and procedure-related adverse events. Longitudinal models can be specified as multi-state models, competing risk models, landmark analyses, or joint longitudinal–survival models that predict transitions from cCR to locoregional regrowth, salvage surgery, or distant metastasis. Such models need at minimum temporal validation, geographic validation, and cross-device validation, and they need prospective impact studies showing whether they reduce missed residual disease, raise biopsy hit rate, detect regrowth earlier, and preserve the opportunity for R0 salvage resection. Deployment also requires arrangements for human review, escalation of abnormal outputs, model drift monitoring, informed patient consent, and defined boundaries of multidisciplinary responsibility.

Progress from model development to clinical use is best achieved in stages, and the stages are not optional. The first involves retrospective development, followed by external validation across centers and devices. The second runs prospective silent validation, assessing data completeness, calibration, technical failure, and workflow fit without influencing treatment decisions. The third compares endoscopists or multidisciplinary teams with and without AI assistance in terms of residual detection rate, false-negative rate, biopsy hit rate, examination time, and changes in management recommendations. The fourth evaluates the lead time gained in detecting residual disease or regrowth, the feasibility of salvage therapy, non-beneficial invasive examinations, quality of life, resource use, and cost-effectiveness. The clinical interface should display prediction uncertainty, the modality contributing most to a given output, and cases with inadequate image quality or out-of-distribution features, so that the AI output functions as a risk signal prompting review and escalation rather than as an automatic treatment instruction [39,41].

Organ preservation research also includes the randomized NEEDS trial (NCT04460352), which compares NCRT followed by planned surgery with definitive chemoradiotherapy followed by surveillance and salvage surgery as needed in operable ESCC. Its treatment contrast differs from SANO’s surveillance-versus-surgery decision after NCRT and cCR. The published protocol provides research context, not outcome evidence or validation of endoscopic AI [57].

## 8. Conclusions

Endoscopy remains the central tool for assessing the primary tumor bed after NCRT in esophageal cancer, and on its own it is not sufficient. Post-treatment inflammation and fibrosis sit on top of residual disease that is deep, scattered, or discontinuous, so excluding tumor on the basis of a single visual inspection and superficial biopsies is unreliable. Persistent or newly appearing focal nodules, irregular ulceration, progressive stricture, and change in lesion extent are the candidate endoscopic indicators worth quantifying. Adequate systematic deep sampling of the primary tumor bed, together with EUS-guided sampling of suspicious lymph nodes, remains the most reliable route to higher sensitivity in clinical response assessment today.

Existing endoscopic AI studies establish that pathologic response can be predicted from pretreatment or post-treatment images, but the only NCRT-specific model uses pretreatment images, and no dedicated post-NCRT image validation is available among the included reports; most studies rest on retrospective static images. The proposed roles for AI are as a second reader for endoscopy, a prompt toward suspicious regions, an aid to biopsy, and a tool for dynamic risk stratification; it cannot independently confirm pCR or decide whether surgery should be omitted. The next step is concrete: move from curated static images to standardized complete video and patient-level longitudinal trajectories, and use both surgical pathology and active surveillance cohorts, image–pathology spatial registration, cross-center external validation, and prospective clinical utility trials to determine whether AI genuinely reduces missed residual disease, avoids surgery that confers no benefit, and protects the window for R0 salvage resection. An AI-assisted system should also be able to recognize inadequate image quality and out-of-distribution cases, report calibrated residual risk together with its uncertainty, and demonstrate incremental value over current multidisciplinary assessment in silent validation and human–AI collaboration studies.

Two things follow for anyone reading this in clinic. First, no model described here has been shown to let a patient skip surgery, and preSINO demonstrates residual diagnostic uncertainty: its 13.5% FNR concerns high-risk lesions, whereas its NPV of 68.7% concerns any residual locoregional disease. Neither is an AI performance estimate nor a surgery-omission threshold. Second, the most informative study still unperformed is not another AUC comparison: it is a prospective trial adding AI-targeted sampling to systematic bite-on-bite biopsy, with the patient-level false-negative rate for residual disease as the primary endpoint. Until such a trial reports its findings, the honest summary is short. Endoscopic AI has the potential to standardize image interpretation, but prospective reader studies are needed to establish reproducibility in practice. Whether it makes active surveillance safer is not yet known.

## Figures and Tables

**Table 1:** Selected endoscopic findings and sampling limitations relevant to response assessment.

Finding and Source	Selected Evidence and Target	Implication for AI-Supported Assessment
Suspicious residual tumor [33]	Residual disease at 6/12 weeks after NCRT: sensitivity 36%/22%; specificity 88%/100%; NPV 29%/33%.	Use focal suspicious findings as sampling alerts; a negative visual impression does not exclude residual disease.
Residual mass or infiltrative/obstructive change [32,33]	Cho categories 3–4: 91.3% non-pCR. Mass, villous surface, and focal traction were frequent among visually suspicious, pathology-positive cases.	Prioritize localization and targeted sampling; the frequency in selected positive cases is not population-level diagnostic sensitivity.
Ulcer [32,33]	Ulcer alone at 6/12 weeks: sensitivity 55%/40%; specificity 39%/57% for residual disease.	Quantify margin, nodularity, and healing over time; ulcer persistence alone is nonspecific.
Stricture and scar [33]	Non-traversable stricture at 6 weeks: 5/11 patients had pCR. Scar NPV for residual disease: 22%/25% at 6/12 weeks.	Assess progression and examination completeness; neither stricture nor a healed surface settles residual status.
Combined visual features [36]	220 ESCC patients after nCT/nCIT; prospective cohorts *n* = 62. Logistic model reported accuracy 96.77% (95% CI 88.83–99.61%).	Indirect evidence only. Manually coded features and same-center prospective validation do not establish post-NCRT AI performance.
Biopsy and nodal sampling [22,23,24,25,26,34,35]	preSINO: NPV 68.7% for any residual disease; high-risk lesion FNR 13.5%, with different endpoint denominators.	Combine systematic tumor-bed biopsy with targeted sampling and suspicious node assessment. AI cannot inspect unsampled tissue.

Table 1. Note: This condensed table retains clinically informative examples rather than a complete inventory of every reported estimate. The target condition and assessment time are specified where diagnostic metrics are given. Results are not pooled or ranked across cohorts. Evidence from nCT/nCIT is indirect for NCRT; scars or normal-appearing mucosa cannot establish patient-level ypT0N0. PPV, positive predictive value; NPV, negative predictive value; IPCL, intraepithelial papillary capillary loop.

**Table 2:** Endoscopy-based response models grouped by clinical task and endpoint.

Study and Setting	Clinical Task and Endpoint	Input and Reference Standard	Validation and Selected Performance	Applicability and Main Limitation
A1 Kawahara 2022 [13] 98 ESCC; NCRT	Pretreatment prediction of reported pCR. Nodal component not clearly specified.	Pretreatment endoscopy; postoperative pathological response.	Abstract: 72 training/26 test; internal cross-validation; mean AUC 0.83, accuracy 80.6%. Full text contains conflicting split counts.	NCRT cohort, but no post-NCRT image detection. No independent external validation; ypT0N0 not established.
A2 Miura 2025 [16] 170 ESCC; NAC	Pretreatment prediction of broader pathologic response (Grade 1b–3 vs. 0–1a), not pCR.	Pretreatment WLE plus clinical variables; surgical regression grade.	Internal patient-level split (reported 85:15) and cross-validation. AUC: image 0.55; clinical 0.64; fused 0.77.	Indirect. Different task and endpoint from both pCR identification and current residual detection.
B1 Matsuda single-center 2023 [14] 193 ESCC; NAC	Post-NAC classification of endoscopic response, not pathological pCR.	Selected post-NAC images; endoscopic responder definition.	20 newly collected test images (10 responders/10 non-responders). Reported sensitivity 60%, specificity 100%, NPV 71%.	Indirect. Subjective morphological target; small balanced test. Patient-level residual exclusion not established.
B2 Matsuda four-center 2023 [15] 123 ESCC; NAC	Post-NAC classification of primary site pCR (Grade 3).	Selected post-NAC images; surgical primary site pathology.	20-photo test; median sensitivity 60%, specificity 81%, NPV 67%, accuracy 70%.	Indirect. Artificial response-group balance; nodes outside endpoint; multicenter collection does not establish center-held-out validation.
B3 Matsuda 46-center 2025 [17] 1041 ESCC; NAC	Post-NAC classification of primary site pCR (Grade 3).	Selected post-NAC WLE; surgical primary site pathology.	Full report describes a 100-photo test. Reported sensitivity 80%, specificity 90%, NPV 82%, accuracy 85%.	Indirect. Selected/balanced response groups; center-held-out validation and patient-level aggregation not verified.
B4 Yuan structured model 2025 [36] 220 ESCC; nCT/nCIT	Post-treatment classification of primary site pCR; nodes explicitly excluded.	Six manually coded visual features ± biopsy; surgical esophageal specimen.	112 training/46 internal validation; 23 + 39 prospective same-center cases. Prospective accuracy 96.77%; AUC 0.99 (95% CI 0.95–1.00).	Indirect; logistic feature model rather than automated image AI. Optimal cut-off used combined cohorts, limiting independent threshold validation.
B5 Yuan EC-HAENet 2025 [37] 300 patients; 297 non-radiotherapy regimens, 3 radiotherapy	Post-treatment classification of primary site pCR/non-pCR; no separately validated NCRT subgroup.	7359 WLE images; surgical primary site pathology.	209 development/91 temporal-test patients; patient-level five-fold split. Test image AUC 0.99; patient accuracy 0.93 (95% CI 0.86–0.98).	Indirect. Same-center temporal test; other patient-level metrics are not used to infer residual disease safety because endpoint orientation/reporting requires reconciliation.

Table 2 note: Groups A and B are pretreatment prediction and post-treatment classification, respectively; no included report separately validates post-NCRT image-based residual detection or AI prediction of surveillance regrowth. Primary site pCR is not ypT0N0. Model performance is interpreted only within its task, target definition, timing, analysis unit, and reference standard. Sensitivity/NPV for a pCR-positive label must not be read as sensitivity/NPV for residual disease detection. Kawahara’s pCR report does not clearly establish a nodal component; it is not treated as validated patient-level ypT0N0. Multicenter data collection is not equivalent to a center-held-out test. Source inconsistencies and inaccessible technical details are identified in the supplement rather than resolved by assumption.

## Data Availability

No new data were created or analyzed in this study. All data discussed are available in the publications cited.

## Supplementary Materials

The following supporting information can be downloaded at: https://www.mdpi.com/article/10.3390/diagnostics16213598/s1, Methods S1: Database-specific search strategies; Table S1A: Endoscopic acquisition and annotation; Table S1B: Model construction and validation; Table S2: Reproducibility and resource availability; Table S3: Independent physician risk of bias and applicability assessments; Table S4: Included reports and evidence category allocation; Figure S1: Independent physician assessments; Figure S2: Flow of report selection. All materials are provided in a single consolidated supplementary file. References cited in the supplementary materials are included in the main-text bibliography [1,2,3,4,5,6,7,8,9,10,11,12,13,14,15,16,17,18,19,20,21,22,23,24,25,26,27,28,29,30,31,32,33,34,35,36,37,38,39,40,41,42,43,44,45,46,47,48,49,50,51,52,53,54,55,56,57].

## Abbreviations

ADC: apparent diffusion coefficient; AI, artificial intelligence; AUC, area under the receiver operating characteristic curve; BLI, blue laser imaging; cCR, clinical complete response; CRE, clinical response evaluation; ctDNA, circulating tumor DNA; ESCC, esophageal squamous cell carcinoma; EUS, endoscopic ultrasonography; EUS-FNA, EUS-guided fine-needle aspiration; IPCL, intraepithelial papillary capillary loop; LCI, linked color imaging; NAC, neoadjuvant chemotherapy; NBI, narrow-band imaging; NCRT, neoadjuvant chemoradiotherapy; nCIT, neoadjuvant chemoimmunotherapy; NPV, negative predictive value; pCR, pathologic complete response; PPV, positive predictive value; SANO, Surgery As Needed for Oesophageal Cancer; TRG, tumor regression grade; TNT, total neoadjuvant therapy; WLE, white-light endoscopy.
