# Supplementary material for: Artificial Intelligence-Assisted Endoscopic Assessment of Response to Neoadjuvant Chemoradiotherapy in Esophageal Cancer: From Residual Disease Detection to Active Surveillance and Surgical Decision-Making
Source: Diagnostics (Basel). 2026 Oct 31;16(21):3598. doi: 10.3390/diagnostics16213598 (PMC13761626; doi:10.3390/diagnostics16213598)
Supplement: Supplementary file 1 [file diagnostics-16-03598-s001.zip › diagnostics-4613511-supplementary.pdf]

Supplementary material

Supplementary Methods S1

PubMed

Search coverage: database inception to 15 July 2026. Record yields below precede deduplication; 244 duplicates were removed across the combined search results.

Main search   Records retrieved 14

("esophageal neoplasms"[MeSH Terms] OR "esophageal cancer"[tiab] OR "esophageal squamous cell carcinoma"[tiab] OR ESCC[tiab] OR "esophageal adenocarcinoma"[tiab])

AND ("neoadjuvant therapy"[MeSH Terms] OR "chemoradiotherapy"[MeSH Terms] OR "neoadjuvant chemoradiotherapy"[tiab] OR NCRT[tiab] OR "neoadjuvant chemotherapy"[tiab] OR NAC[tiab] OR "total neoadjuvant therapy"[tiab])

AND ("endoscopy"[MeSH Terms] OR "endoscopic ultrasonography"[MeSH Terms] OR endoscopy[tiab] OR EUS[tiab] OR "endoscopic biopsy"[tiab] OR "narrow band imaging"[tiab] OR NBI[tiab])

AND ("artificial intelligence"[MeSH Terms] OR "machine learning"[MeSH Terms] OR "deep learning"[MeSH Terms] OR "neural networks, computer"[MeSH Terms] OR "artificial intelligence"[tiab] OR "deep learning"[tiab] OR "convolutional neural network"[tiab] OR CNN[tiab] OR radiomics[tiab])

Supplementary active-surveillance search   Records retrieved 104

("esophageal neoplasms"[MeSH Terms] OR "esophageal cancer"[tiab])

AND (NCRT[tiab] OR "neoadjuvant chemoradiotherapy"[tiab])

AND ("active surveillance"[tiab] OR "watchful waiting"[tiab] OR "salvage esophagectomy"[tiab] OR "clinical complete response"[tiab] OR cCR[tiab])

Supplementary Methods S1

Embase

Main search   Records retrieved 58

#1 'esophagus tumor'/exp OR 'esophageal cancer':ti,ab,kw OR 'oesophageal cancer':ti,ab,kw OR 'esophageal squamous cell carcinoma':ti,ab,kw OR 'oesophageal squamous cell carcinoma':ti,ab,kw OR ESCC:ti,ab,kw OR 'esophageal adenocarcinoma':ti,ab,kw OR 'oesophageal adenocarcinoma':ti,ab,kw

#2 'neoadjuvant chemotherapy'/exp OR 'chemoradiotherapy'/exp OR 'neoadjuvant chemoradiotherapy':ti,ab,kw OR 'neoadjuvant chemoradiation':ti,ab,kw OR NCRT:ti,ab,kw OR 'neoadjuvant chemotherapy':ti,ab,kw OR NAC:ti,ab,kw OR 'total neoadjuvant therapy':ti,ab,kw

#3 'endoscopy'/exp OR 'endoscopic ultrasonography'/exp OR 'endoscopic biopsy'/exp OR 'narrow band imaging'/exp OR endoscop*:ti,ab,kw OR EUS:ti,ab,kw OR 'endoscopic biopsy':ti,ab,kw OR 'narrow band imaging':ti,ab,kw OR NBI:ti,ab,kw

#4 'artificial intelligence'/exp OR 'machine learning'/exp OR 'deep learning'/exp OR 'convolutional neural network'/exp OR 'radiomics'/exp OR 'artificial intelligence':ti,ab,kw OR 'machine learning':ti,ab,kw OR 'deep learning':ti,ab,kw OR 'convolutional neural network*':ti,ab,kw OR CNN:ti,ab,kw OR radiomics:ti,ab,kw

#5 #1 AND #2 AND #3 AND #4

Supplementary active-surveillance search   Records retrieved 134

#6 'esophagus tumor'/exp OR 'esophageal cancer':ti,ab,kw OR 'oesophageal cancer':ti,ab,kw

#7 NCRT:ti,ab,kw OR 'neoadjuvant chemoradiotherapy':ti,ab,kw OR 'neoadjuvant chemoradiation':ti,ab,kw

#8 'active surveillance':ti,ab,kw OR 'watchful waiting':ti,ab,kw OR 'salvage esophagectomy':ti,ab,kw OR 'salvage oesophagectomy':ti,ab,kw OR 'clinical complete response':ti,ab,kw OR cCR:ti,ab,kw

#9 #6 AND #7 AND #8

Supplementary Methods S1

Cochrane Library

Combined search   Records retrieved 38

#1 [mh "Esophageal Neoplasms"] OR ("esophageal cancer" OR "oesophageal cancer"):ti,ab,kw OR ("esophageal squamous cell carcinoma" OR "oesophageal squamous cell carcinoma"):ti,ab,kw OR ESCC:ti,ab,kw OR ("esophageal adenocarcinoma" OR "oesophageal adenocarcinoma"):ti,ab,kw

#2 [mh "Neoadjuvant Therapy"] OR [mh Chemoradiotherapy] OR ("neoadjuvant chemoradiotherapy" OR "neoadjuvant chemoradiation"):ti,ab,kw OR NCRT:ti,ab,kw OR "neoadjuvant chemotherapy":ti,ab,kw OR NAC:ti,ab,kw OR "total neoadjuvant therapy":ti,ab,kw

#3 [mh Endoscopy] OR [mh Endosonography] OR endoscop*:ti,ab,kw OR EUS:ti,ab,kw OR "endoscopic biopsy":ti,ab,kw OR ("narrow band imaging" OR "narrow-band imaging"):ti,ab,kw OR NBI:ti,ab,kw

#4 [mh "Artificial Intelligence"] OR [mh "Machine Learning"] OR [mh "Deep Learning"] OR [mh "Neural Networks, Computer"] OR "artificial intelligence":ti,ab,kw OR "machine learning":ti,ab,kw OR "deep learning":ti,ab,kw OR "convolutional neural network*":ti,ab,kw OR CNN:ti,ab,kw OR radiomics:ti,ab,kw

#5 #1 AND #2 AND #3 AND #4

Table S1A Endoscopic acquisition and annotation

Study	Dataset and centers	Histology and treatment	Modality and image/video input	Preprocessing	Annotation
Kawahara [13]	98 patients; 1 center; total original-image count not clearly established	ESCC; NCRT	Pretreatment static endoscopic images; no video validation	JPEG; 150×150; Laplacian/Sobel/Haar-wavelet filters; augmentation	Patient pCR/non-pCR labels; no spatial tumor annotation established
Matsuda single-center [14]	193 development patients; 1 center; 20 new test images	ESCC; NAC	Post-NAC static endoscopy; two representative images per patient	227 × 227 RGB images; MATLAB R2018a/AlexNet input	Endoscopic responder/non-responder; morphology-based target
Matsuda four-center [15]	123 patients; 4 centers; 20-photo test	ESCC; NAC; response groups balanced by selection	Post-NAC static endoscopy; two representative images per patient	227 × 227 RGB images; MATLAB R2022b/AlexNet input	Surgical primary-site Grade 3 vs non-pCR; class labels; spatial annotations not described in full text
Miura [16]	170 patients; 1384 images in submitted extraction; 1 center	ESCC; NAC	Pretreatment WLE plus clinical/laboratory variables	NV in the technical-information extraction	Surgical response Grade 1b–3 vs 0–1a; image-level spatial annotation NV
Matsuda 46-center [17]	1041 patients; 46 centers; 1861 development images reported; 100-image test	ESCC; NAC; selected response-group balance	Post-NAC WLE still images; video not evaluated	224 × 224 RGB; test-image rotations (−90° to +90°) and X/Y reflections reported	Surgical primary-site Grade 3 vs non-pCR; one test image per patient; spatial annotations not described
Yuan structured model [36]	220 patients; 1 center; retrospective 158, prospective 62	ESCC; nCT/nCIT	Post-treatment endoscopy; manually coded visual features ± biopsy	Binary feature coding; biopsy has three categories	Two endoscopists assess six visual features; third resolves disagreement; surgical primary-site pCR labels
Yuan EC-HAENet [37]	300 patients; 1 center; 7359 images (4561/2798)	ESCC or adenocarcinoma eligible; nCT/nCIT 297; radiotherapy 3	Post-treatment WLE; two temporal cohorts; no real-time video	ImageNet pretraining; random augmentation; focal loss; sharpness-aware minimization; hyperparameters in appendix	Patient and images assigned surgical primary-site pCR/non-pCR; no lesion-pathology spatial gold standard

Technical entries for Matsuda [14], [15] and [17] were checked against the supplied full-text PDFs; separate article supplements were not independently checked. NV denotes a technical item not verified in this extraction, not absence of full-text risk-of-bias appraisal. Rows represent reports, not necessarily independent cohorts. Reference numbers follow the manuscript.

Table S1B Model construction and validation

Study	AI architecture	Training/validation/test split	External validation	Analysis unit	Reference standard
Kawahara [13]	16-layer CNN with filtering; Adam	Abstract 72/26; full-text prediction section says 72/38; five-fold CV. Inconsistency retained explicitly.	None independent	Patient partition stated; reported averages from internal CV	Postoperative pCR; ypN requirement not clearly documented
Matsuda single-center [14]	AlexNet; MATLAB R2018a; final three layers replaced for binary ER classification	Training:validation 7.5:1; 20 newly collected test images (10 ER/10 non-ER), one per patient	New test images not used for development; no held-out-center validation established	Image classification; one image per test patient; multi-image aggregation not specified	Endoscopic response classification; not surgical pCR
Matsuda four-center [15]	AlexNet; MATLAB R2022b; binary pCR classification; 20 fitted models	123 selected response-balanced cases; training:validation 9:1; 20 test images (10 pCR/10 non-pCR)	Independent test images; full text states that development/test centers were not completely separated	Image classification; one image per test patient; multi-image aggregation not specified	Surgical Grade 3 of primary lesion; nodal disease not part of target
Miura [16]	ResNet50 image/clinical fusion; clinical comparator logistic regression	85:15 patient split and internal CV in submitted extraction; fold details NV	None independent in submitted extraction	Patient model; image aggregation details NV	Surgical pathological response Grade 1b–3 vs 0–1a
Matsuda 46-center [17]	ResNet-50; MATLAB R2023b; Adam; 50 epochs; learning rate 0.0001; batch size 64	Image-level 9:1 shuffle-split; 100 test images from 100 patients excluded from development	46-center collection; held-out-center validation not established; internal patient separation not specified	Image classification; one image per test patient; multi-image aggregation not specified	Surgical primary-site Grade 3 vs non-pCR
Yuan structured model [36]	Multivariable logistic regression/nomogram; six-feature sensitivity analysis	112 train/46 internal; repeated five-fold CV; 23+39 prospective. Cut-off optimized across cohorts.	Prospective temporal/same-center cohorts; no center-held-out test	Patient; manually coded endoscopic features	Resection primary-site pCR; nodes explicitly excluded
Yuan EC-HAENet [37]	DenseNet-121 local branch + Swin Transformer global branch; ensemble classifier; XGrad-CAM	209 development patients with patient-level five-fold CV; 91 temporally independent test patients	Temporal same-center validation; no held-out center	Image and patient; any image classified non-pCR makes patient non-pCR	Surgical primary-site pCR; no residual tumor at primary site, irrespective of nodal clearance

Technical entries for Matsuda [14], [15] and [17] were checked against the supplied full-text PDFs; separate article supplements were not independently checked. NV denotes a technical item not verified in this extraction, not absence of full-text risk-of-bias appraisal. Rows represent reports, not necessarily independent cohorts. Reference numbers follow the manuscript.

Table S2 Reproducibility and resource availability

Study	Dataset access	Source code	Trained weights	Preprocessing and replication information
Kawahara [13]	No public dataset link identified in accessible full text	No public link identified	No public link identified	Filters, image size and CNN training described; inconsistent split counts prevent exact reconstruction
Matsuda single-center [14]	No public patient-image dataset link identified in reviewed full text	No study-specific code link identified in reviewed full text	No study-trained weights link identified in reviewed full text	Full text, PDF p. 3: AlexNet/MATLAB R2018a; two representative images per patient; 227 × 227 RGB; training:validation ratio 7.5:1; 20 newly collected test images; threshold 0.5. No executable study pipeline identified in the full text.
Matsuda four-center [15]	No public patient-image dataset link identified in reviewed full text	No study-specific code link identified in reviewed full text	No study-trained weights link identified in reviewed full text	Full text, PDF pp. 3–4: AlexNet/MATLAB R2022b; two representative images per patient; 227 × 227 RGB; training:validation ratio 9:1; 20 test images; threshold 0.5. The report states that development and test centers were not completely separated.
Miura [16]	Reasonable request to corresponding author (publisher statement)	Public code link NV	Public weights link NV	ResNet50/fusion verified; full preprocessing and aggregation NV
Matsuda 46-center [17]	No public patient-image dataset link identified in reviewed full text	UNPIC visualization-app link reported; no study-specific training/inference code link identified in reviewed full text	No study-trained weights link identified in reviewed full text	Full text, PDF pp. 2–4: ResNet-50/MATLAB R2023b; 224 × 224 RGB; 9:1 shuffle-split; Adam, 50 epochs, learning rate 0.0001, batch size 64; 100 test images. Test-image rotation/reflection is reported. No patient-level split list identified in the full text.
Yuan structured model [36]	No downloadable patient dataset identified in accessible article	No executable repository identified	Not a neural-weight model; published coefficients/nomogram available	Feature definitions, coding and model coefficients reported; exact independent threshold validation remains limited
Yuan EC-HAENet [37]	Article/supplement; further inquiries to corresponding authors; no public patient-image archive identified	No public code repository identified in accessible article	No downloadable trained weights identified	Architecture/training details in appendices; article-level description does not establish a reproducible executable package

"No public link identified" refers only to reviewed text, not confirmed unavailability. Generic software is distinguished from study-specific model code. NV means not verified. Other entries were retained. This resource audit is separate from Table S3 risk-of-bias appraisal. Reports may share cohorts; numbering follows the manuscript.

Table S3 Independent physician risk of bias and applicability assessments

Domain judgments from two independent physicians. All paired risk-of-bias and applicability judgments agreed. L = Low; U = Unclear; H = High.

Risk domains: D1 Participants and data sources; D2 Predictors; D3 Outcome; D4 Analysis. Applicability concerns are shown separately for D1-D3.

A Prediction models (PROBAST+AI 2025)

Tool publication: main-text reference [20].

Study / assessment	Risk D1	Risk D2	Risk D3	Risk D4	Applicability D1	Applicability D2	Applicability D3
M01 Kawahara 2022 [13]	U	U	U	U	L	L	U
M02 Matsuda 2023 single centre [14]	U	U	U	U	L	L	U
M03 Matsuda 2023 four centres [15]	U	U	U	U	L	L	L
M04 Miura 2025 [16]	U	U	U	U	L	L	L
M05 Matsuda 2025 46 centres [17]	U	U	U	U	L	L	L
M06 Yuan 2025 structured model [36]	U	U	L	U	L	L	L
M07 Yuan 2025 EC-HAENet [37]	U	U	U	U	L	L	L
M08 Gangaram Panday 2026 [55]	U	U	U	U	L	U	L

B Conventional diagnostic accuracy (QUADAS-2)

Study / assessment	Risk D1	Risk D2	Risk D3	Risk D4	Applicability D1	Applicability D2	Applicability D3
D01 preSINO Yang 2025 [22]	L	U	U	U	L	L	L
D02 preSANO Noordman 2018 [25]	L	U	U	U	L	L	L
D03 van der Bogt 2021 [33]	H	L	U	H	L	L	L
D04 Cho 2018 [32]	L	U	U	H	L	L	L
D05 Taghizadeh Kermani 2022 [35]	H	U	U	H	L	L	L
D06 Khaitan 2022 [34]	U	U	U	H	L	L	L
D07 van der Bogt 2020 EUS estimate [21]	H	L	U	H	L	L	L
D08 van der Bogt 2020 FNA estimate [21]	H	L	U	H	L	L	L
D09 van der Bogt 2019 EUS measurements [26]	L	U	U	U	L	L	L

PROBAST+AI 2025 model-evaluation risk of bias is reported; model-development quality is not presented as a separate completed appraisal. Gangaram Panday is the additional routine-clinical-variable model. The other seven rows are core endoscopy-based modelling reports.

The model analysis domain was Unclear in all eight reports. There were 31 Unclear and one Low judgment across the 32 model domains; these counts are descriptive and are not a quality score.

Nine assessment rows correspond to eight reports; D07 and D08 share the same article and DOI. Four patient-selection and six flow-and-timing judgments were High; all nine reference-standard judgments were Unclear. Reports derived from shared cohorts are not independent replications.

Scope limitation: SANO has signaling-question records only, without final domain judgments, and is excluded from this matrix. A complete cluster-randomized RoB 2 appraisal was not performed. ROBINS-I was not used; non-randomized management comparisons and background-only sources were not formally appraised. Reference numbers follow the revised manuscript.

Table S4 Included reports and evidence category allocation

Forty-nine reports were assigned once by primary contribution: A, 1; B, 21; C, 6; D, 21. Categories indicate relevance to the review question, not certainty of evidence. Reference numbers match the revised manuscript.

Reference and report	Group	Primary contribution	DOI
[1] Bondzi-Simpson, A. et al. 2024	D	Decision-analytic comparison of surveillance and surgery	10.1016/j.jtcvs.2024.04.020
[2] Smyth, N.M. et al. 2026	D	Clinical outcomes of surveillance or surgery	10.1016/j.ejso.2025.111171
[3] Theeuwen, H. et al. 2023	D	Clinical review of surveillance and salvage surgery	10.1002/jso.27182
[4] van der Wilk, B.J. et al. 2021	D	Non-randomized surveillance versus surgery comparison	10.1097/SLA.0000000000003636
[5] van der Zijden, C.J. et al. 2023	D	Prospective active-surveillance research context	10.1186/s12885-023-10747-z
[6] van der Wilk, B.J. et al. 2025	D	Randomized surveillance versus surgery comparison	10.1016/S1470-2045(25)00027-0
[7] Schneider, P.M. et al. 2008	B	Endoscopy, biopsy and EUS response assessment	10.1097/SLA.0b013e31818f3afb
[8] Ngamruengphong, S. et al. 2010	B	Diagnostic systematic review of EUS and FDG-PET	10.1111/j.1442-2050.2009.00989.x
[9] van Rossum, P.S. et al. 2016	B	Systematic review of endoscopic biopsy and EUS	10.1016/j.gie.2015.11.026
[10] Heneghan, H.M. et al. 2016	B	Clinical PET and endoscopic residual-disease assessment	10.1097/SLA.0000000000001902
[11] de Gouw, D.J. et al. 2019	B	Diagnostic imaging systematic review and meta-analysis	10.1016/j.jtho.2019.04.004
[13] Kawahara, D. et al. 2022	A	Pretreatment endoscopic-image response prediction in NCRT	10.1259/bjr.20210934
[14] Matsuda, S. et al. 2023	C	Post-NAC endoscopic responder classification	10.1245/s10434-023-13140-z
[15] Matsuda, S. et al. 2023	C	Post-NAC primary-site pathological-response model	10.1245/s10434-023-13862-0
[16] Miura, T. et al. 2025	C	Pretreatment endoscopic and clinical NAC-response prediction	10.1007/s10388-025-01106-x
[17] Matsuda, S. et al. 2025	C	Post-NAC primary-site pathological-response model	10.1007/s10388-025-01130-x
[18] Stiles, B.M. et al. 2019	D	Histology and treatment-selection context	10.1016/j.athoracsur.2018.07.089
[19] Bayley, E.M. et al. 2023	D	Clinical and pathological risk features of adenocarcinoma	10.1097/SLA.0000000000005693
[21] van der Bogt, R.D. et al. 2020	B	EUS and FNA detection of residual nodal disease	10.1055/a-1065-1759
[22] Yang, Y. et al. 2025	B	Prospective clinical residual-disease evaluation	10.1093/bjs/znaf004
[23] Chao, Y.K. et al. 2015	B	Factors in false-negative post-treatment biopsy	10.1097/MD.0000000000000588
[24] Chao, Y.K. et al. 2017	B	Post-treatment endoscopic-biopsy accuracy	10.1016/j.ejso.2017.09.022
[25] Noordman, B.J. et al. 2018	B	Prospective clinical residual-disease evaluation	10.1016/S1470-2045(18)30201-8
[26] van der Bogt, R. et al. 2019	B	EUS measurements of residual disease	10.1055/a-0795-3220
[27] Shapiro, J. et al. 2013	B	Residual-tumor distribution in mucosa and submucosa	10.1097/SLA.0b013e3182a6191d
[28] Chao, Y.K. et al. 2015	B	Pathology underlying false-negative endoscopic biopsies	10.1245/s10434-015-4412-8
[29] Chao, Y.K. et al. 2016	B	Distribution of near-complete-response residual tumor	10.1002/bjs.10293
[30] Chao, Y.K. et al. 2018	B	Anatomical distribution of residual cancer	10.1093/ejcts/ezx261
[31] van der Wilk, B.J. et al. 2020	B	Residual sites missed by endoscopic biopsies	10.1002/bjs.11760
[32] Cho, C.J. et al. 2018	B	Endoscopic categories of chemoradiotherapy response	10.1111/jgh.14055
[33] van der Bogt, R.D. et al. 2021	B	Endoscopic findings predictive of residual disease	10.1055/a-1362-9375
[34] Khaitan, P.G. et al. 2022	B	Clinical versus pathological response	10.1007/s11605-022-05315-y
[35] Taghizadeh Kermani, A. et al. 2022	B	Endoscopic observation and biopsy performance	10.3389/fonc.2022.859079
[36] Yuan, P. et al. 2025	C	Post-nCT/nCIT structured endoscopic-feature model	10.1055/a-2625-5884
[37] Yuan, P. et al. 2025	C	Post-treatment endoscopic AI in a predominantly non-radiotherapy cohort	10.3389/fonc.2025.1590448
[42] Hu, X. et al. 2026	D	CT-based longitudinal prognosis modelling after definitive CRT	10.7150/jca.126804
[43] Yu, N. et al. 2025	D	CT-based locoregional-recurrence modelling after definitive CRT	10.3390/cancers17010126
[44] Liu, Y. et al. 2024	D	Multimodal radiomics for pathological response	10.1186/s13244-024-01851-0
[45] Vollenbrock, S.E. et al. 2020	B	MRI added to conventional endoscopic/endosonographic assessment	10.1007/s00330-019-06605-x
[46] Zhang, M. et al. 2024	D	CT radiomics and hematological response biomarkers	10.1016/j.tranon.2023.101804
[47] Liu, Y. et al. 2024	D	MR radiomics and dynamic hematological response factors	10.1016/j.heliyon.2024.e33702
[48] Beukinga, R.J. et al. 2021	D	PET radiomics and tissue-marker response modelling	10.1007/s00330-020-07439-8
[49] Zhang, C. et al. 2020	D	Pretreatment biopsy immune-signature context	10.1038/s41392-020-00221-8
[50] Liu, Z. et al. 2025	D	ctDNA detection of residual disease and adjuvant-treatment context	10.1016/j.xcrm.2025.102334
[51] Qi, B. et al. 2025	D	Radiopathomics after neoadjuvant chemoimmunotherapy	10.1136/jitc-2025-013840
[52] Chen, Y. et al. 2024	D	Histopathology-based response and survival modelling	10.1016/j.saa.2024.124030
[53] Wu, W.Z. et al. 2025	D	Serum multiomics after concurrent chemoradiotherapy	10.1038/s41416-025-03229-5
[55] Gangaram Panday, S.S.G. et al. 2026	D	Routine-clinical-variable response/progression prediction	10.1245/s10434-025-18531-y
[56] Gerber, S. et al. 2026	D	Clinical predictors of recurrence during watchful waiting	10.3390/curroncol33030170

A uses pretreatment images from an NCRT-treated cohort. C comprises indirect models, including one predominantly non-radiotherapy cohort with three radiotherapy-treated patients and no separate validated NCRT subgroup.

Eight additional technical, methodological or protocol citations [12,20,38–41,54,57] are outside the 49-report selection and are not counted in the flow diagram. Reports may share cohorts, and reviews may summarize the same primary studies. The 16 formally appraised reports are a subset of the 49, not an additional inclusion total.

Figure S1 Independent physician assessments

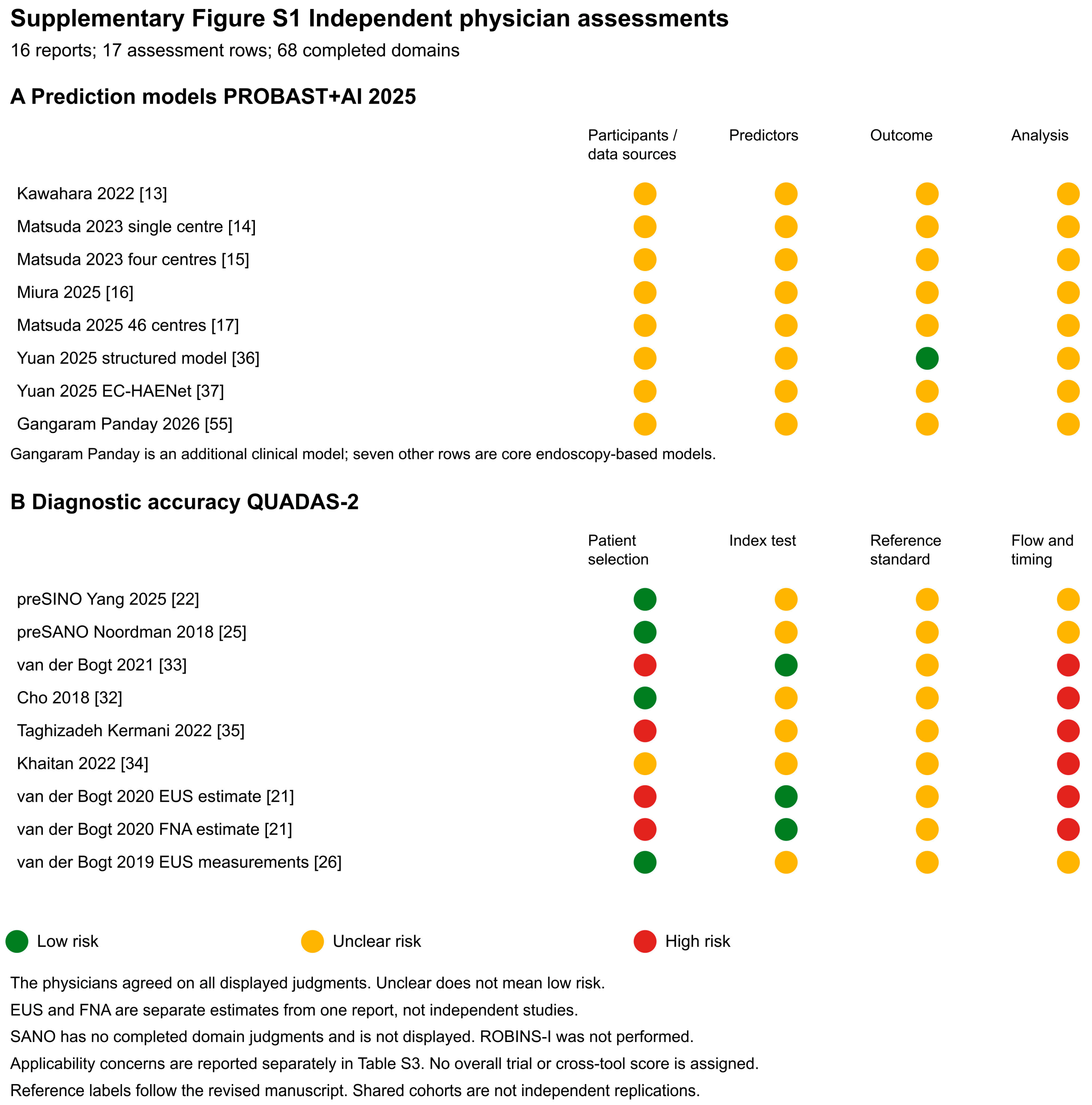

Completed risk-of-bias judgments from two independent physicians. PROBAST+AI tool publication: main-text reference [20].

Figure S2 Flow of report selection

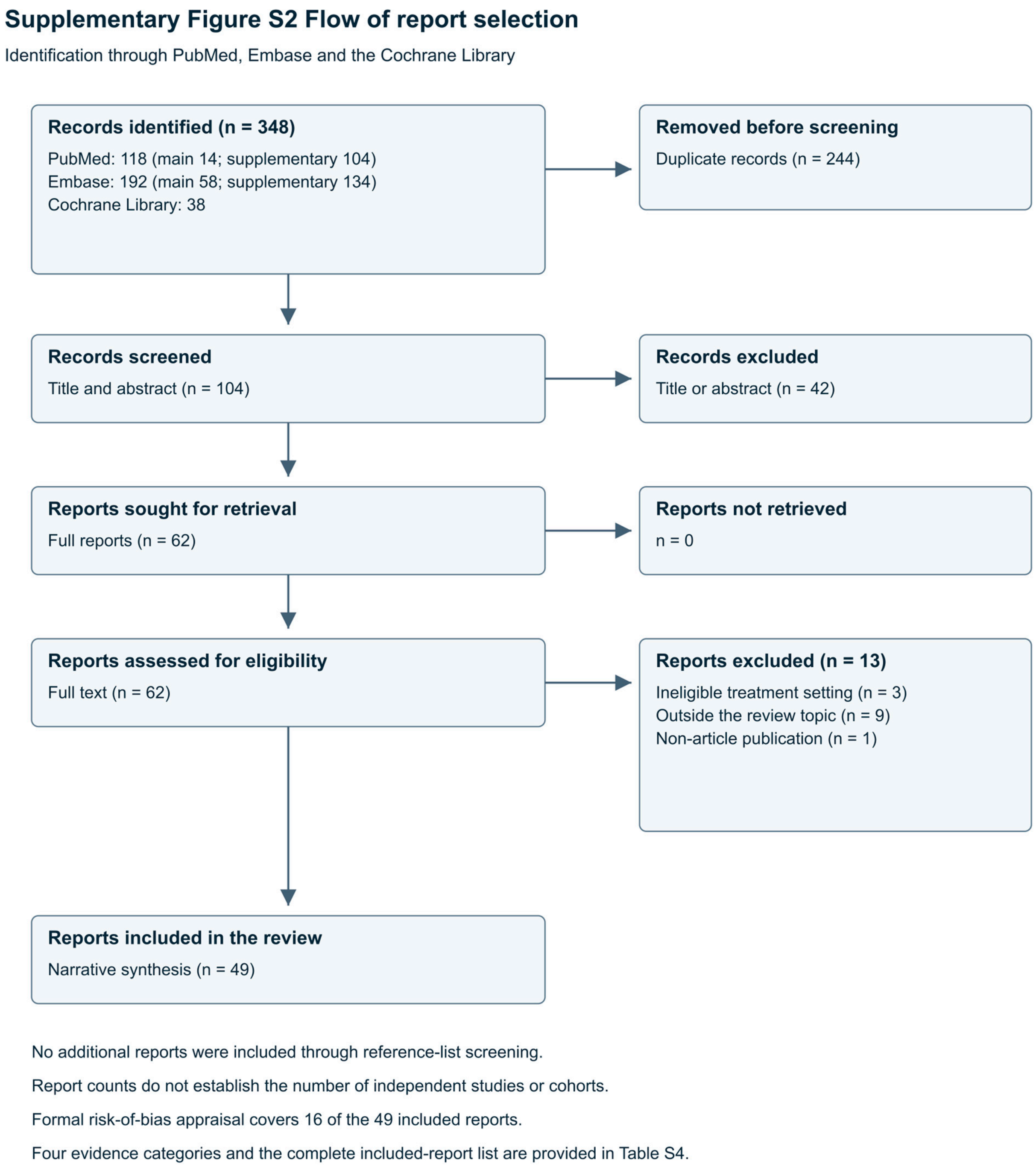

Report selection from PubMed, Embase and the Cochrane Library. The 49 included reports are listed in Table S4.
